# ^13^C-^13^C Homonuclear Recoupling in Solid-State Nuclear Magnetic Resonance at a Moderately High Magic-Angle-Spinning Frequency

**DOI:** 10.1371/journal.pone.0050504

**Published:** 2013-01-10

**Authors:** Venus Singh Mithu, Subha Bakthavatsalam, Perunthiruthy K. Madhu

**Affiliations:** Department of Chemical Sciences, Tata Institute of Fundamental Research, Homi Bhabha Road, Colaba, Mumbai, India; National Institute for Medical Research, Medical Research Council, United Kingdom

## Abstract

Two-dimensional ^13^C-^13^C correlation experiments are widely employed in structure determination of protein assemblies using solid-state nuclear magnetic resonance. Here, we investigate the process of ^13^C-^13^C magnetisation transfer at a moderate magic-angle-spinning frequency of 30 kHz using some of the prominent second-order dipolar recoupling schemes. The effect of isotropic chemical-shift difference and spatial distance between two carbons and amplitude of radio frequency on ^1^H channel on the magnetisation transfer efficiency of these schemes is discussed in detail.

## Introduction

**Figure 1:**
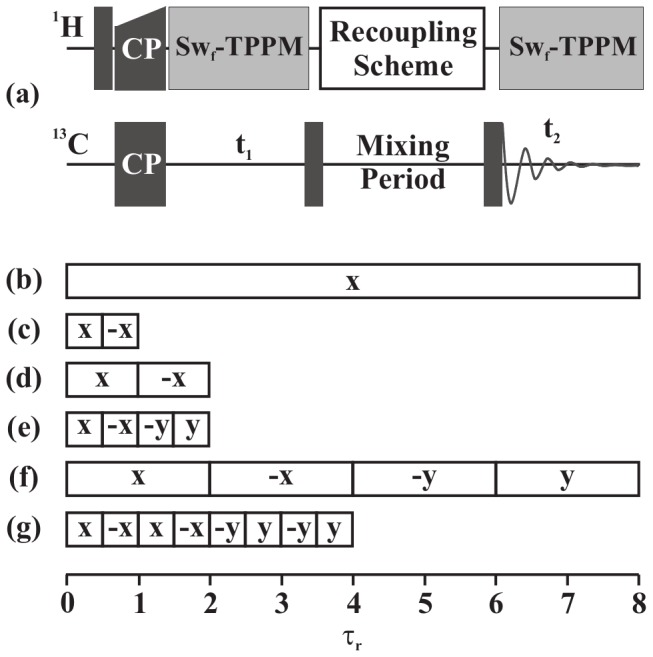
Schematic of recoupling pulse sequence and blocks. (a) General pulse scheme for a 2D ^13^C-^13^C spin-diffusion experiment. Second-order recoupling schemes (b) DARR, (c) PARIS (N = 1/2), (d) PARIS (N = 2), (e) PARIS-xy (m = 1)(N = 1/2), (f) PARIS-xy (m = 1)(N = 2), and (g) PARIS-xy (M = 2)(N = 1/2) are applied on the ^1^H channel during the mixing period.

Technical advances in the past decade have made solid-state nuclear magnetic resonance (SSNMR) a powerful tool in the field of structural biology, especially for systems like protein assemblies and membrane proteins [1]–[5]. With the advent of various two- and three-dimensional (2D and 3D) experiments, the structural information obtained with SSNMR has become more precise and reliable [5]–[9]. Structure information in SSNMR is mostly derived from the less abundant spins like ^13^C and ^15^N, with through-space 2D ^13^C-^13^C correlation experiments playing a prominent role [7]. Through-space ^13^C-^13^C correlations can be obtained with first-order homonuclear dipolar recoupling schemes [10]–[14]. But these schemes suffer from dipolar truncation effects where strong dipolar couplings mask the weaker ones [15]. Whilst this effect helps in the selective observation of directly bonded carbon atoms, which is very helpful in assigning the ^13^C resonances in a protein skeleton, the same effect makes these schemes unsuitable for observing long-range ^13^C-^13^C contacts, which are essential for determining tertiary structure of proteins. A number of recoupling schemes with attenuated dipolar truncation are available today [14], [16]–[20]. Second-order dipolar recoupling schemes are one such kind of schemes and have been widely used to observe long range ^13^C-^13^C correlations [21], [22]. Proton Driven Spin Diffusion (PDSD) [23], Dipolar Assisted Rotational Resonance (DARR) [24], Rf-Assisted Diffusion (RAD) [25], Phase Alternated Recoupling Irradiation Schemes (PARIS [26], [27] and PARIS-xy [28]), and the recently introduced Second-order Hamiltonian among Analogous Nuclei Generated by Heteronuclear Assistance Irradiation (SHANGHAI) [29] come under the category of second-order recoupling schemes. Figure 1 shows the pulse sequence for obtaining a 2D ^13^C-^13^C dipolar correlation spectrum and a schematic representation of some of the recoupling schemes mentioned above.

**Figure 2:**
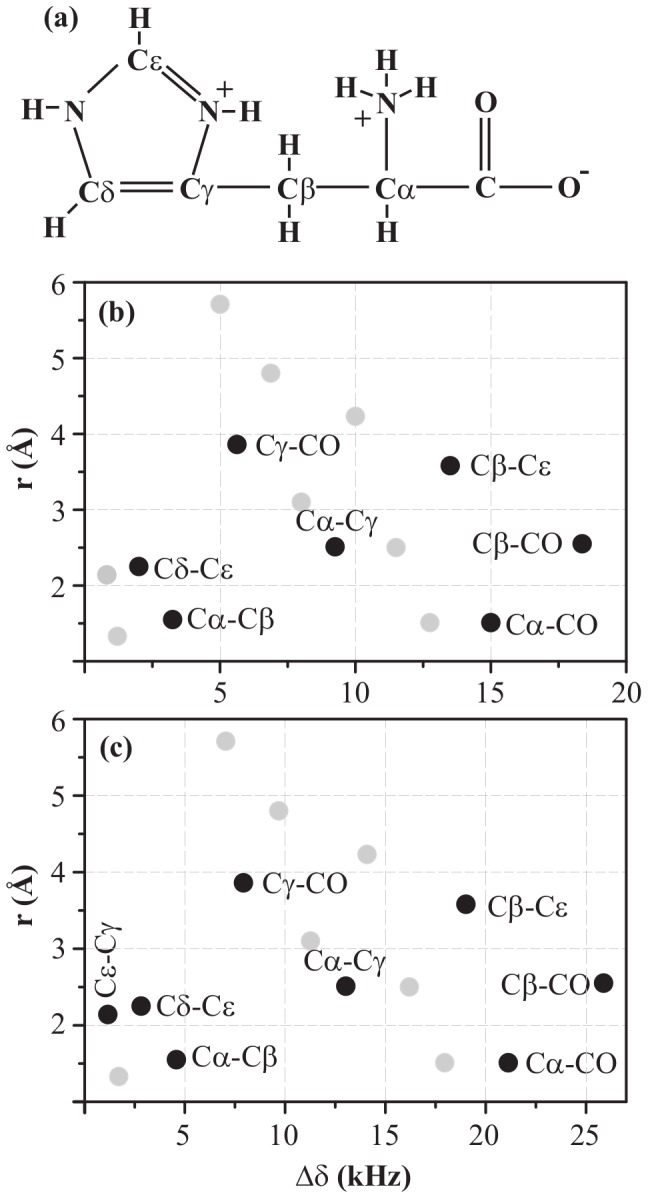
2D ^13^C-^13^C spin-diffusion spectrum. (a) A schematic molecular structure of histidine molecule. A map of all possible cross-peaks in a 2D ^13^C-^13^C spin-diffusion spectrum of U-^13^C-,^15^N-L-histidine

Efficiency of magnetisation transfer between two carbon nuclei during a recoupling experiment using a second-order recoupling scheme is strongly dependent on the following: (1) Spatial distance (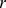) between them (spatial proximity), (2) Isotropic chemical-shift difference (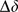) between them (chemical proximity), and (3) Strength of ^1^H- ^1^H dipolar couplings present in the proton bath around them. An ideal recoupling scheme should be insensitive to changes in these factors. Such an ideal scheme is not available till date because the last two factors are highly sensitive to changes in the experimental conditions. The second factor depends on the magnitude of external magnetic field (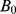). Low 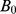 values lead to small 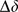 values which are good for these recoupling schemes. This is primarily because all types of cross-peaks are spread over a few kHz, thus not putting the broad-banded nature of any recoupling scheme under harsh test. (The spread of all possible cross-peaks of histidine at two different 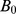 values, 11.74 and 16.43 T corresponding to ^1^H Larmor frequency of 500 and 700 MHz is shown in Figure 2.) Secondly, because all chemical-shift anisotropy (CSA) spinning side-bands can be moved out of the main spectrum by using low magic-angle-spinning (MAS) frequencies (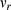). Maximum 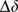 value in a 2D ^13^C-^13^C spectrum of a normal protein is not more than 170 ppm (approximately 21 kHz at 11.74 T and 30 kHz at 16.43 T). That means in order to avoid any interference from the spinning side-bands, one should ideally stick to 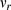 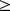
21 at 11.74 T and 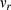 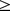
30 kHz at 16.43 T. Moreover, low spinning frequencies also ensure a strongly coupled ^1^H- ^1^H bath which again is a favourable condition for more efficient spin diffusion [7].

H
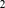
O as a function of chemical-shift difference (along X axis) and spatial distance (along Y axis) between two carbon atoms at magnetic fields of (b) 11.74 and (c) 16.43 T. Highlighted cross-peaks are the ones which are studied in this work.

Everything seems to be perfect about performing 2D ^13^C-^13^C spin diffusion experiments at low magnetic fields except one thing; poor spectral resolution. This is certainly a big problem if one intends to do SSNMR of biomolecules. Whilst high magnetic fields yield high-resolution spectra, they also lead to some other problems. At higher 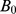 values, the cross-peaks are spread over many kHz (see Figure 2c), thus putting the broad-banded nature of any recoupling sequence under serious test. Moreover, higher spinning frequencies would be required so that spinning side-bands do not interfere with the actual cross-peaks in the main spectrum. This will lead to a weakly coupled ^1^H- ^1^H bath and hence, inefficient spin-diffusion. Longer mixing times (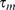) will thus be required to observe sufficient correlations which eventually means that only low RF amplitudes can be applied on the ^1^H channel (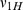) during the mixing time period in order to avoid sample heating [30] due to absorption of energy from RF electric fields. Hence, to obtain a highly resolved 2D ^13^C-^13^C spectrum, one needs a broad-banded recoupling scheme which should perform efficiently at high spinning frequencies using 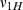 values as low as possible. Recoupling schemes like PARIS, PARIS-xy, and the recently introduced SHANGHAI have been designed to address these issues. These schemes have been shown to work efficiently using low 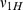 at high spinning frequencies and magnetic fields. These schemes are also found to be more broad-banded, especially SHANGHAI and the PARIS-xy family.

In this work we have studied the magnetisation transfer efficiency of DARR, PARIS (N = 1/2), PARIS (N = 2), PARIS-xy (m = 1)(N = 1/2), PARIS-xy (m = 1)(N = 2), and PARIS-xy (m = 2)(N = 1/2) recoupling schemes as a function of 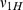 and 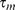 at 30 kHz of spinning frequency at two external magnetic fields; 11.74 T and 16.43 T. We found that even for broad-banded schemes like PARIS-xy (m = 1)(N = 1/2), PARIS-xy (m = 1)(N = 2), and PARIS-xy (m = 2)(N = 1/2), magnetisation transfer efficiencies can be seriously attenuated by a wrong choice of 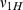. Whilst magnetisation transfer between one type of carbon pair is much better at low 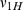 values, yet another pair of carbon nuclei shows maximum magnetisation transfer only when high RF amplitudes are applied on the ^1^H channel. This behaviour is found to be guided mainly by the isotropic chemical-shift difference between the two carbon nuclei, which can vary from very small (e.g. two aliphatic carbons) to very large (e.g. an aliphatic and a carbonyl carbon) in the same protein sample. Here, low RF amplitudes correspond to 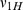 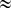 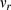/4 and 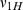 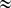 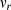/2 at 11.74 and 16.43 T respectively and high RF amplitude correspond to 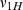 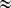 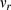. Like 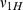, different cross-peaks respond in a different manner to changes in the mixing-time period. These differences are found to be predominantly guided by the spatial distance between the two carbons.

Observations stated above, derived from measurements made on a sample of U-^13^C-,^15^N-L-histidine

H
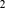
O, are subsequently verified on a sample of U-^13^C-,^15^N-N-Formyl Methionyl-Leucyl Phenylalanine (N-fMLF). We also demonstrate the effect of low and high 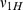 on the 2D ^13^C-^13^C spectrum of the 42 amino acid long peptide Amyloid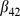 (A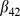), aggregated forms of which are considered to be the reason behind the pathology of Alzheimer's disease [31], [32].

## Results and Discussion

A 2D ^13^C-^13^C spin diffusion spectrum of U-^13^C-,^15^N-L-histidine

H
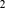
O can consist of fifteen different kinds of cross-peaks at most. These peaks, distributed as a function of chemical-shift difference and spatial distance between the carbons giving rise to cross-peaks, are shown in Figure 2 at magnetic fields of 11.74 and 16.43 T. On the basis of chemical-shift difference, we classify histidine cross-peaks into three classes:

- **Chemically close (** 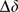 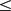 **5 kHz):** Most important cross-peaks in this class are the aliphatic-aliphatic ones. These cross-peaks play a crucial role in chemical-shift assignment of the carbon skeleton of an amino acid. C 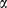 and C 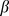 chemical shifts obtained from these peaks help predicting the secondary structure adopted by a particular amino acid in a protein [33]–[35].
- **Chemically apart (5 kHz** 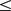 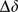 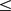 **15 kHz):** Cross-peaks between aliphatic-aromatic and aromatic-carbonyl carbons belong to this class. These cross-peaks are of great help in identifying aromatic amino acids and thus can act as check-points whilst doing chemical-shift assignment of a protein sequence.
- **Chemically far apart (** 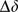 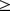 **15 kHz):** This class mainly consists of cross-peaks between aliphatic and carbonyl carbons. The carbonyl chemical shifts generally obtained from these cross-peaks also play a vital role in predicting secondary structure adopted by a protein [33]–[35]. Moreover, these cross-peaks are of great help in assigning amino acids which have a carboxyl group in their side-chains, e.g. Asp and Glu.

We now study the efficiency of magnetisation transfer as a function of 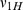 and 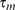 for second-order recoupling schemes, namely, DARR, PARIS (N = 1/2), PARIS (N = 2), PARIS-xy (m = 1)(N = 1/2), PARIS-xy (m = 1)(N = 2), and PARIS-xy (m = 2)(N = 1/2). Magnetisation transfer efficiency (*M*) is calculated using the following equation:
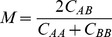
(1)where 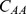 and 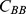 are intensities of two diagonal peaks and 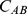 is the intensity of the cross-peak between them [36].

### 1 Effect of 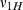 on efficiency of magnetisation transfer

**Figure 3:**
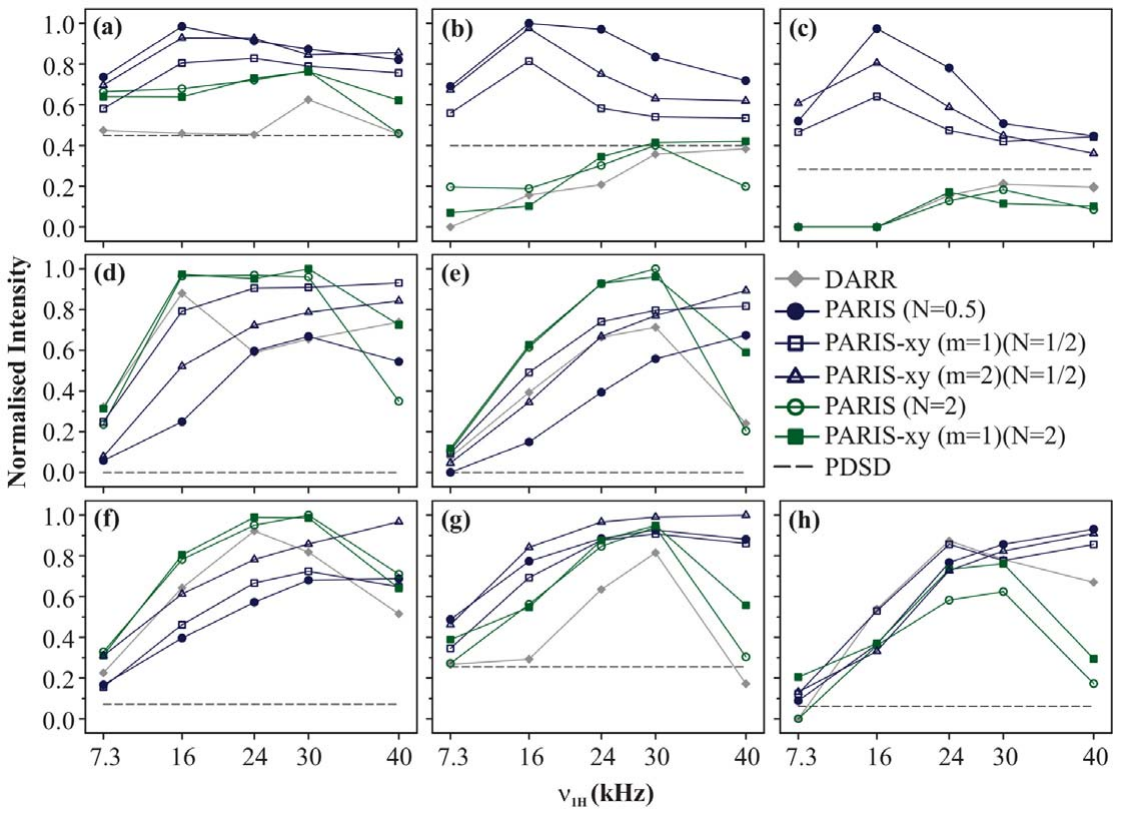
Build-up curves. Magnetisation build-up curves of (a) C 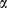-C 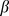, (b) C 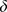-C 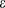, (c) C 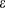-C 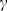, (d) C 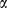-C 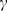, (e) C 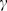-CO, (f) C 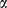-CO, (g) C 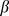-CO, and (h) C 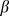-C 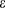 cross-peaks of U-^13^C-,^15^N-L-histidine

Figure 3 shows the magnetisation build-up curves of DARR, PDSD, PARIS (N = 1/2), PARIS (N = 2), PARIS-xy (m = 1)(N = 1/2), PARIS-xy (m = 1)(N = 2), and PARIS-xy (m = 2)(N = 1/2) recoupling schemes obtained for eight different cross-peaks of histidine. All measurements were made at a magnetic field of 16.43 T with 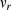 = 30 kHz and 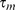 = 90 ms. For a given cross-peak, *M* values are calculated for each recoupling scheme as described in Eq. 1 for five different RF amplitudes. These values are then normalised with respect to the best *M* value obtained for each cross-peak. Black dotted line in each case indicates magnetisation transfer efficiency of PDSD. Figure 3a shows build-up curves for C 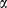-C 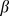 cross-peak. Largest *M* value is yielded by PARIS (N = 1/2) using an RF amplitude of 16 kHz followed by PARIS-xy (m = 2)(N = 1/2). More importantly, these two give their best performance at low RF amplitudes (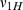 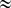 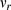/2) on the ^1^H channel. But efficiency significantly drops when 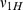 is further lowered to 7.3 kHz (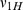 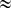 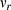/4). PARIS-xy (m = 1)(N = 1/2) also follows a similar trend, but is slightly less efficient in comparison to these two. PARIS (N = 2) and PARIS-xy (m = 1)(N = 2) not only yield small *M* values (largest values 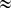
80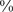 of the maximum value yielded by PARIS (N = 1/2)), but also require high RF amplitudes on the ^1^H channel (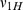 = 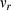) which is not a favourable condition. DARR gives its best performance at rotary-resonance condition (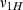 = 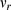) but fairs poorly as compared to other schemes except PDSD. Similar trends are observed for other two chemically-close (class A) cross-peaks, C 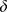-C 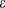 (Figure 3b) and C 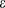-C 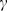 (Figure 3c), but with substantial change in the magnitude of observed values. These differences most likely arise from the relatively large spatial distance between the two carbons in case of C 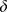-C 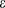 and C 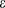-C 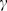 cross-peaks. PARIS (N = 1/2) yields the highest *M* values followed by PARIS-xy (m = 2)(N = 1/2) and PARIS-xy (m = 1)(N = 1/2) using low RF amplitudes (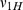 = 16 kHz) for both cross-peaks. DARR, PARIS(N = 2), and PARIS-xy (m = 1)(N = 2) yield very small *M* values and also require high RF amplitudes for optimal performance. Their performance is even worse than PDSD, where no RF is applied at all.

H
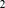
O for different recoupling schemes applied on the ^1^H channel, at 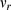 = 30 kHz and 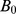 = 16.43 T. Each recoupling scheme was applied for a fixed mixing-time period (90 ms) using different RF amplitudes of 7.3, 16, 24, 30, and 40 kHz. 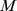 values calculated for individual cross-peaks according to Eq. 1 are plotted as a function of RF amplitude. 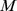 values are normalised with respect to the maximum value yielded by PARIS (N = 1/2) (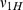 = 16 kHz) in (a, b, and c), PARIS-xy (m = 1)(N = 2) (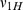 = 30 kHz) in (d), PARIS (N = 2) (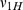 = 30 kHz) in (e and f), PARIS-xy (m = 2)(N = 1/2) (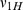 = 30 kHz) in (g), and PARIS (N = 1/2) (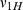 = 40 kHz) in (h). Data points are joined with straight lines to guide the readers' eyes.

Figure 3d shows build-up curves for C 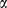-C 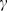 cross-peak (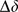 = 13 kHz), a class B (chemically apart) cross-peak. The highest *M* value is yielded by PARIS-xy (m = 1)(N = 2) in this case at 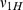 = 30 kHz, followed by PARIS (N = 2) at 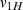 = 16 kHz, PARIS-xy (m = 1)(N = 1/2) at 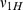 = 40 kHz, and DARR at 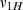 = 16 kHz. All these values are within 6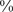 of each other. Here, the performance of PARIS-xy (m = 1)(N = 2) and PARIS (N = 2) is much better than other schemes considering the fact that they are quite insensitive towards changes in 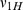. Though these two lose their robustness in case of C 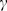-CO cross-peak (class B; Figure 3e), they still yield the largest *M* values among all. Also, as observed in case of class A cross-peaks, DARR, PARIS (N = 2), and PARIS-xy (m = 1)(N = 2) perform best when high RF amplitudes are applied (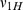 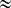 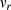). PARIS (N = 1/2), PARIS-xy (m = 1)(N = 1/2), and PARIS-xy (m = 2)(N = 1/2) on the other hand not only yield relatively small *M* values for chemically-apart cross-peaks, they also perform most efficiently only when high RF amplitudes are applied, which is exactly opposite to what was observed in case of chemically-close cross-peaks.

Figure 3f shows build-up curves for C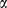-CO cross-peak, a class C (chemically far apart) cross-peak. The highest *M* value in this case is yielded by PARIS (N = 2) using a RF amplitude of 30 kHz. PARIS-xy (m = 1)(N = 2) and DARR also perform very well in this case. Build-up curve of PARIS-xy (m = 1)(N = 2) is nearly identical to that of PARIS (N = 2). DARR gives its best performance at an intermediate RF amplitude of 24 kHz and not at the rotary-resonance condition. Trends observed for two other chemically far apart cross-peaks, C 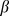-CO and C 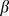-C 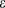, are shown in Figures 3g and 3h, respectively. In case of C 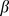-CO, largest *M* value is yielded by PARIS-xy (m = 2)(N = 1/2) (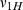 = 40 kHz). Maximum *M* values yielded by other recoupling sequences (except DARR) are within 10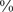 of each other. In case of C 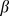-C 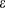 largest *M* value is yielded by PARIS (N = 1/2) (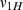 = 40 kHz) and maximum *M* values yielded by other recoupling schemes (except PARIS (N = 2)) are within 12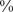 of each other. Like in the case of chemically-apart cross-peaks, here also, all recoupling schemes require high RF amplitudes to perform better. Thus, in general, we can say that except for chemically-close cross-peaks, one requires high RF amplitudes (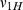 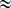 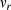) for efficient magnetisation transfer for all recoupling schemes, which is not a favourable condition for protein samples.

All these observations tell us that the profile of build-up curves is strongly dependent on chemical-shift difference between the two carbons giving rise to a particular cross-peak. A comparison between build-up curves of C 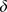-C 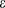 (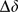 = 4.6 kHz; Figure 3b), C 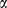-C 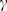 (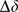 = 13.0 kHz; Figure 3d), and C 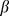-CO (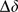 = 25.8 kHz; Figure 3g) cross-peaks which differ minimally in terms of spatial distance but enormously in terms of 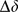 makes this point clearer. Therefore, we expect that a 2D ^13^C-^13^C spectrum recorded at a moderately high spinning frequency of, say, 30 kHz, using DARR, PARIS (N = 2), or PARIS-xy (m = 1)(N = 2) recoupling scheme will yield poor cross-peak intensities in the aliphatic region which can be even poorer than those obtained using a simple PDSD recoupling scheme. Longer mixing times could be helpful here, but we will see in the next section that it helps only when the carbons are spatially far apart from each other. And since these schemes perform better only when high RF amplitudes are applied, use of long mixing times can cause sample heating and should be avoided. Another way of increasing peak intensities is by recording more number of scans per free induction decay. But this would increase the total experimental time and, hence, is certainly not a preferred solution. Therefore, DARR, PARIS (N = 2), or PARIS-xy (m = 1)(N = 2) recoupling schemes are not very favourable whilst recording a 2D ^13^C-^13^C spectrum meant for chemical-shift assignment of aliphatic carbons in a protein sample at moderately high spinning frequencies. PARIS (N = 1/2) would be an excellent choice in such scenarios as it is not only efficient in this region, but also requires low RF amplitudes for optimal performance. Its major drawback is that it yields poor intensities for all other cross-peaks even when high RF amplitudes are applied. One way to get rid of this problem would be to record a spectrum using an intermediate RF amplitude of, say, 24 kHz (used in this study). At this amplitude, intensity of class A cross-peaks would certainly decrease, but would still be more than what can be obtained using other schemes. On the other hand, one would gain substantial intensity for class B and C cross-peaks. But if one has to make this compromise at all, using PARIS-xy (m = 1)(N = 1/2) or PARIS-xy (m = 2)(N = 1/2) recoupling scheme would be a better option. These two are much more broad-banded in nature as compared to PARIS (N = 1/2) as shown earlier also [28]. These two schemes not only give a fairly good performance in aliphatic region, but also perform much better than PARIS (N = 1/2) in other regions, thus making them ideal candidates for obtaining a 2D ^13^C-^13^C spectrum with fairly good cross-peak intensities in all regions. But a true broad-banded recoupling scheme should yield equally good cross-peak intensities in all regions using low RF amplitudes, a condition, which unfortunately, none of the aforementioned schemes satisfies in true sense.

**Figure 4:**
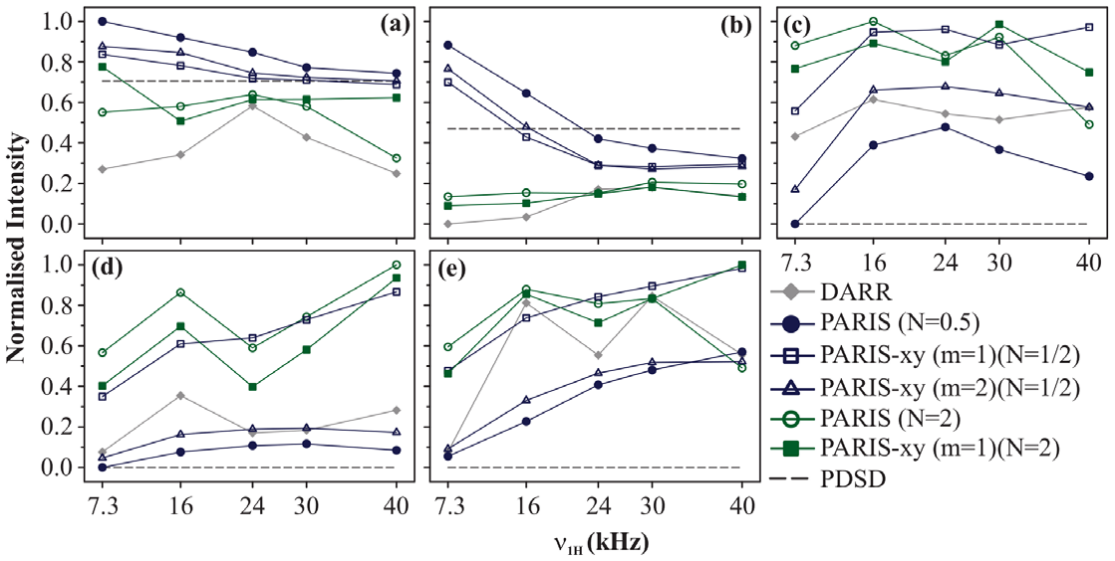
Build-up curves. Magnetisation build-up curves of (a) C 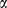-C 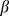, (b) C 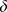-C 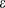, (c) C 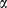-C 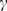, (d) C 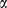-CO, and (e) C 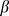-CO cross-peaks of U-^13^C-,^15^N-L-histidine

We now discuss the magnetisation build-up curves obtained at a magnetic field of 11.74 T at 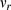 = 30 kHz with 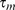 = 90 ms. Figures 4a and 4b show the build-up curves for chemically-close cross-peaks C 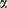-C 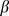 and C 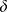-C 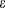, respectively. In both cases, PARIS (N = 1/2) yields the largest *M* value followed by PARIS-xy (m = 2)(N = 1/2) and PARIS-xy (m = 1)(N = 1/2) using an RF amplitude of just 7.3 kHz. DARR, PARIS (N = 2), and PARIS-xy (m = 1)(N = 2) not only yield very small *M* values but also require higher RF amplitudes to perform optimally. These observations are very similar to the ones observed at 16.43 T, except that here magnetisation transfer efficiency of PARIS (N = 1/2), PARIS-xy (m = 2)(N = 1/2), and PARIS-xy (m = 1)(N = 1/2) does not drop even when a very low RF amplitude of 7.3 kHz is applied. This is most likely because the 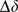 values for these cross-peaks have changed (reduced in this case) as the external magnetic field is lowered. Figure 4c shows the build-up curves for C 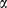-C 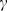 (class B cross-peak). In this case, PARIS(N = 2), PARIS-xy(m = 1)(N = 2), and PARIS-xy(m = 1)(N = 1/2) not only yield large *M* values than the rest of the three schemes, they do so using a low RF amplitude of 16 kHz. Figure 4d shows the build-up curves for C 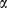-CO which, in this case, is a class B cross-peak with 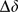 value of 15 kHz. Largest *M* value is yielded by PARIS(N = 2) followed by PARIS-xy (m = 1)(N = 2) and PARIS-xy (m = 1)(N = 1/2). PARIS(N = 2) does show reasonably good performance using a low RF amplitude of 16 kHz. All other schemes in this case deliver a poor performance. Magnetisation build-up profiles observed in case of C 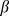-CO (Figure 4e) are quite similar to C 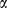-CO except that here DARR also performs reasonably well.

H
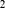
O for different recoupling schemes applied on the ^1^H channel, at 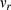 = 30 kHz and 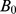 = 11.74 T. Each recoupling scheme was applied for a fixed mixing-time period (90 ms) using different RF amplitudes of 7.3, 16, 24, 30, and 40 kHz. 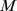 values calculated for individual cross-peaks according to Eq. 1 are plotted as a function of RF amplitude. 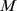 values are normalised with respect to the maximum value yielded by PARIS (N = 1/2) (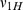 = 7.3 kHz) in (a and b), PARIS (N = 2) (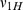 = 16 kHz) in (c), PARIS (N = 2) (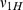 = 40 kHz) in (d), and PARIS-xy (m = 1)(N = 2) (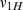 = 40 kHz) in (e). Data points are joined with straight lines to guide the readers' eyes.

**Figure 5:**
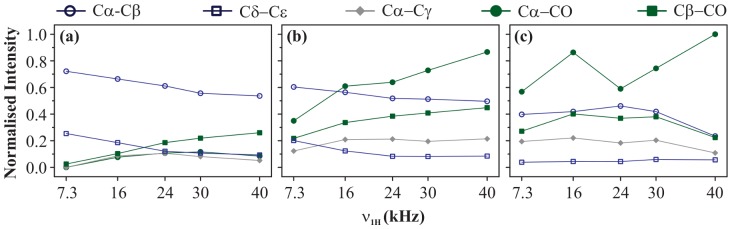
Build-up curves. Magnetisation build-up curves obtained using (a) PARIS (N = 1/2), (b) PARIS-xy (m = 1)(N = 1/2), and (c) PARIS (N = 2) recoupling schemes for different cross-peaks of U-^13^C-,^15^N-L-histidine

Magnetisation build-up curves obtained at the lower magnetic field certainly look different from those observed at the higher field, especially for chemically-apart and far-apart cross-peaks. Here also PARIS (N = 2) and PARIS-xy (m = 1)(N = 2) yield good intensities but only for chemically-apart and far-apart cross-peaks. An advantage which they have here is that they perform much better even at low RF amplitudes (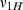 = 16 kHz) for these kind of cross-peaks. But in case of chemically-close cross-peaks, their performance is even worse than the simple PDSD scheme. For such peaks, PARIS (N = 1/2) yields high intensities and that too using extremely low RF amplitudes. But it is a relatively inefficient scheme when it comes to chemically-apart and far-apart cross-peaks even if one uses high RF amplitudes. PARIS-xy (m = 2)(N = 1/2) looses its broad-banded nature at lower magnetic field and displays magnetisation build-up curves which are very similar to that of PARIS (N = 1/2). PARIS-xy (m = 1)(N = 1/2) on the other hand not just maintains, but actually improves its broad-banded nature at lower magnetic field. It satisfies all criteria of an ideal recoupling scheme at this moderately high spinning frequency to a large extent. It is broad-banded, requires low RF amplitude on the ^1^H channel to do so, and also yields fairly good cross-peak intensities as compared to the best values obtained using other regio-specific schemes. Figure 5 illustrates this point in a much better fashion. In this figure the magnetisation build-up curves are displayed for (a) PARIS (N = 1/2), (b) PARIS-xy (m = 1)(N = 1/2), and (c) PARIS (N = 2) obtained for the aforementioned cross-peaks at 11.74 T. All *M* values (calculated according to Eq. 1) are normalised with respect to the largest *M* value obtained for C 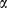-CO cross-peak using PARIS (N = 2) recoupling scheme at an RF amplitude of 40 kHz. Unlike PARIS (N = 1/2) and PARIS (N = 2), PARIS-xy (m = 1)(N = 1/2) yields similar *M* values for chemically-close (C 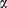-C 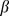) and chemically-apart (C 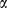-CO) cross-peaks using an RF amplitude of just 16 kHz. The reason why other three cross-peaks (C 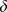-C 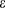, C 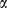-C 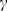, and C 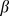-CO) yield lower *M* values as compared to C 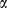-C 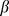 and C 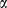-CO is not the ineffective broad-banded nature of PARIS-xy (m = 1)(N = 1/2), but the carbons giving rise to these cross-peaks are spatially more distant (see Figure 2b). We will discuss the effect of spatial distance on magnetisation transfer in the next section. This is an important point that one should keep in mind whilst doing such comparison studies.

H
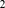
O, at 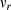 = 30 kHz and 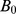 = 11.74 T. Each recoupling scheme was applied for a fixed mixing-time period (90 ms) using different RF amplitudes of 7.3, 16, 24, 30, and 40 kHz. 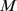 values calculated for individual cross-peaks according to Eq. 1 are plotted as a function of RF amplitude. All 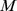 values are normalised with respect to the maximum value yielded by PARIS (N = 2) using a RF amplitude of 40 kHz. Data points are joined with straight lines to guide the readers' eyes.

**Figure 6:**
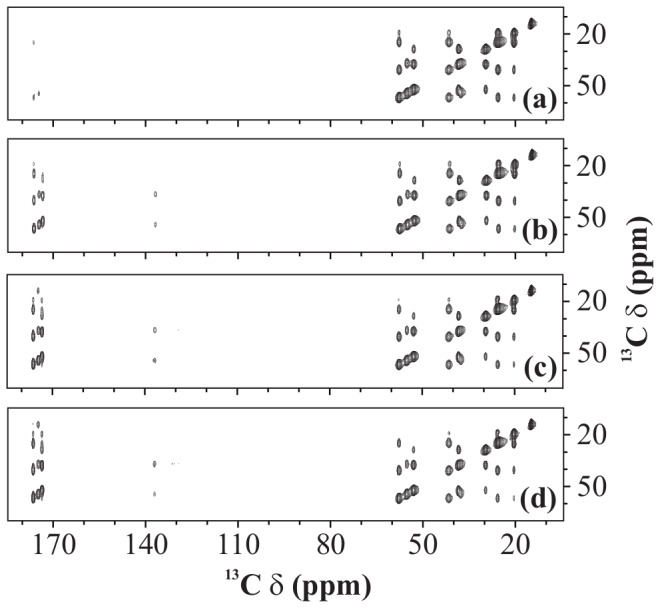
^13^C-^13^C spectra. 2D ^13^C-^13^C spectra of U-^13^C-,^15^N-fMLF recorded using PARIS-xy (m = 1)(N = 1/2) recoupling scheme at MAS frequency of 30 kHz and an external magnetic field of 16.43 T. Mixing-time period in each case was 90 ms. Different proton RF amplitudes of (a) 7.3 kHz, (b) 16 kHz, (c) 24 kHz, and (d) 30 kHz were employed. Only a selected region of 2D spectrum, covering cross-peaks from each class, is shown in each case.

We now demonstrate these observations on a more complex system, U-^13^C-,^15^N-fMLF. Figure 6 shows a set of 2D ^13^C-^13^C spectra of U-^13^C-,^15^N-fMLF recorded using PARIS-xy (m = 1)(N = 1/2) recoupling scheme with different RF amplitudes at a magnetic field of 16.43 T. It is clear from these spectra that as the 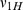 value reaches the rotary-resonance condition (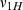 = 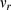), the class (A) cross-peaks (aliphatic-aliphatic) loose intensity, whilst the class (B) (aliphatic-aromatic), and class C (aliphatic-carbonyl) cross-peaks become more and more intense. This is totally in line with what was observed in case of U-^13^C-,^15^N-L-histidine

**Figure 7:**
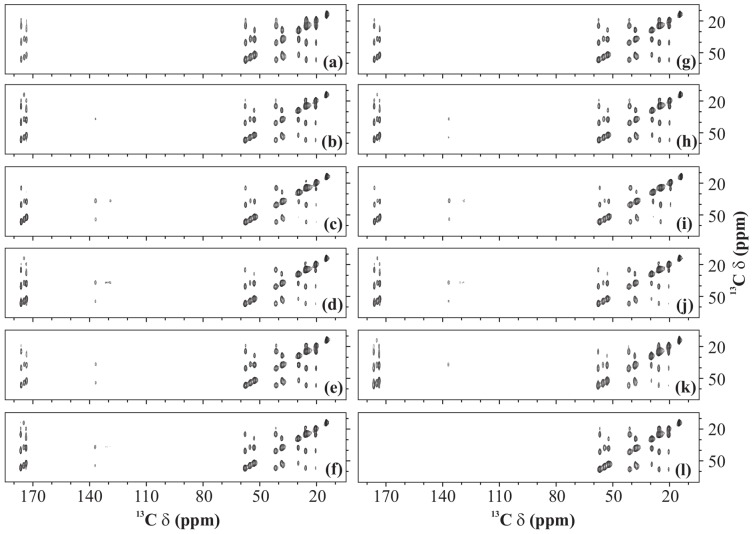
^13^C-^13^C spectra. 2D ^13^C-^13^C spectra of U-^13^C-,^15^N-fMLF recorded with (a and b) PARIS (N = 1/2), (c and d) PARIS (N = 1/2), (e and f) (e) PARIS-xy (m = 1)(N = 1/2), (g and h) PARIS-xy (m = 1)(N = 2), (i and j) PARIS-xy (M = 2)(N = 1/2), (k) DARR, and (l) PDSD recoupling scheme using an RF amplitude of 16 kHz in (a, c, e, g, and i), 30 kHz in (b, d, f, h, j, and k) and without RF in (l). Each spectrum was recorded at a MAS frequency of 30 kHz, magnetic field of 16.43 T, and using a 90 ms long mixing-time period.

H
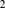
O. Figure 7 shows selected region of U-^13^C-,^15^N-fMLF 2D ^13^C-^13^C spectra recorded using various recoupling schemes with 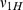 = 16 kHz (a, c, e, g, and i) and 30 kHz (b, d, f, h, j, and k). In all cases, spectra recorded using a low RF amplitude yield more intense cross-peaks in the aliphatic region whilst those recorded using a high RF amplitude yield more intense cross-peaks in the aromatic and carbonyl regions. A PDSD spectrum (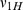 = 0 kHz) is also shown for reference (Figure 7(l)).

**Figure 8:**
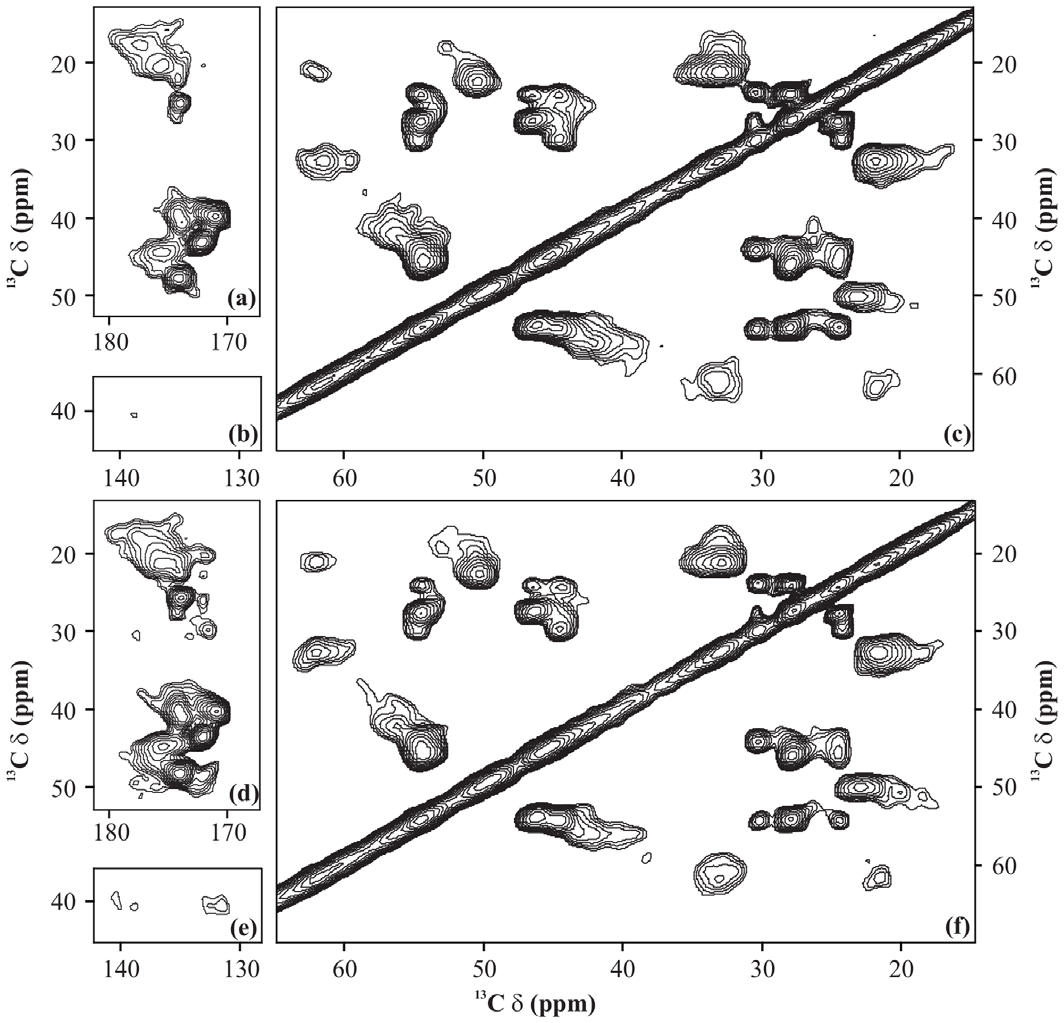
^13^C-^13^C spectra. 2D ^13^C-^13^C spectra of A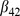 recorded at 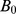 = 16.43 T and 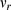 = 30 kHz. PARIS-xy (m = 1)(N = 1/2) recoupling scheme was employed during a 90 ms long mixing-time period using RF amplitudes of (a, b, c) 16 and (d, e, f) 30 kHz. Selected regions of 2D spectrum, covering (a, d) aliphatic-carbonyl, (b, e) aliphatic-aromatic, and (c, f) aliphatic-aliphatic cross-peaks, are shown in both the cases.

Figure 8 shows 2D ^13^C-^13^C spectra of A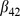 recorded at 16.43 T, 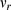 = 30 kHz using PARIS-xy(m = 1)(N = 1/2) with an RF amplitude of 16 (Figure 8(a, b, and c)) and 30 (Figure 8(d, e, and f)) kHz. As expected, the aliphatic-carbonyl and aliphatic-aromatic cross-peaks are more intense when 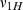 = 30 kHz (Figures 8d and 8e) than at 16 kHz (Figures 8a and 8b). Aliphatic-aliphatic cross-peaks on the other hand loose intensity when the RF amplitude on the ^1^H channel is increased from 16 kHz (Figure 8c) to 30 kHz (Figure 8f), but this drop in intensity is less prominent as compared to the gain in intensity observed in other regions. Hence, it is quite clear that lower RF amplitudes should be employed if one is “only” interested in cross-peaks in the aliphatic region. For all other cross-peaks, higher RF amplitudes and short mixing-time periods seem to be the most reasonable option.

### 2 Effect of 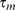 on efficiency of magnetisation transfer

**Figure 9:**
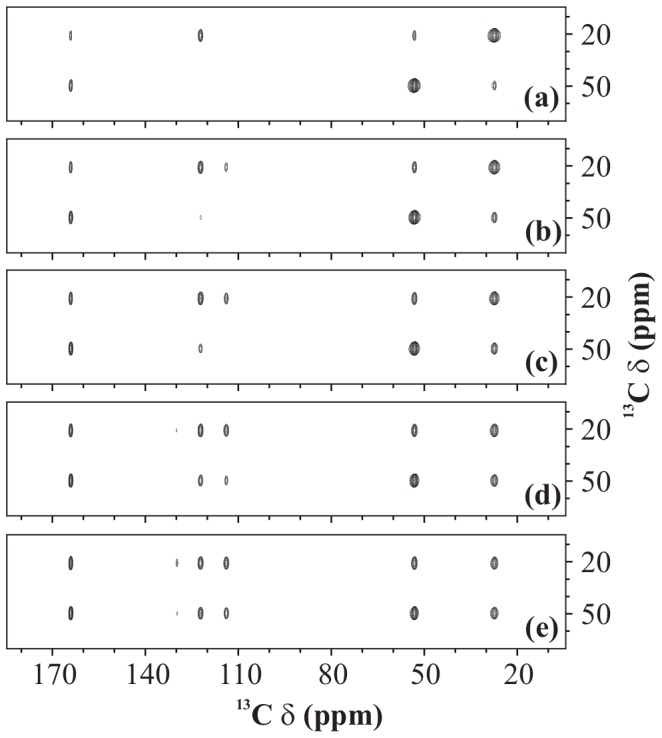
^13^C-^13^C spectra. 2D ^13^C-^13^C spectrum of U-^13^C-,^15^N-L-histidine

Mixing time becomes a very crucial factor when a second-order recoupling scheme is used to obtain a 2D ^13^C-^13^C spectrum. It gives the experimentalist a handle over the type of interaction that is being observed. For example, in a spectrum recorded with a very short mixing-time period, one can see cross-peaks between only those carbons which are spatially very close, like directly-bonded carbons. This kind of spectrum is really helpful in assigning the protein backbone. Once the assignment is done, longer mixing times can be used to observe long-range ^13^C-^13^C interactions. Figure 9 shows the effect of duration of the mixing-time period on the 2D ^13^C-^13^C spectrum of U-^13^C-,^15^N-L-histidine

**Figure 10:**
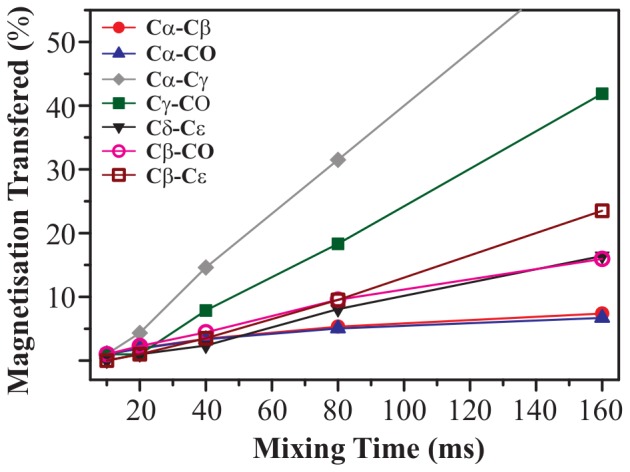
Mixing-time effect. Effect of mixing-time period on magnetisation transfer efficiency for different cross-peaks of U-^13^C-,^15^N-L-histidine

H
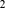
O recorded using PARIS-xy (m = 1)(N = 1/2) recoupling scheme at 30 kHz of spinning frequency using an RF amplitude of 30 kHz at 11.74 T of magnetic field. Quite clearly, all cross-peaks become more intense at longer mixing times, but some cross-peaks experience much drastic changes as compared to others. These changes are presented in a more quantitative fashion in Figure 10 for some selective cross-peaks; C 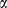-C 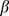 (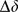 = 3.25 kHz, 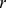 = 1.55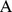), C 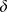-C 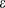 (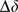 = 2.00 kHz, 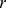 = 2.25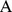), C 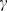-CO (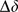 = 5.63 kHz, 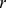 = 3.86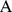), C 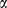-C 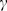 (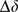 = 9.25 kHz, 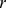 = 2.51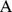), C 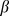-C 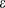 (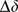 = 13.5 kHz, 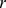 = 3.58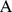), C 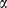-CO (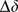 = 15.00 kHz, 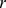 = 1.51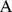), and C 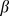-CO (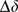 = 18.38 kHz, 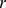 = 2.55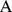). This gives an exact picture of intensity changes experienced by a cross-peak when mixing time is increased from 10 to 160 ms.

H
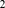
O recorded using PARIS-xy (m = 1)(N = 1/2) recoupling scheme at 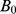 = 11.74 T, 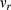 = 30 kHz, 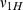 = 16 kHz, and with (a) 10, (b) 20, (c) 40, (d) 80, and (e) 160 ms long mixing-time period.

H
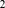
O at 11.74 T. For each cross-peak, 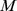 values were calculated for each mixing time using Eq. 1. All 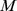 values obtained for a particular cross-peak are normalised with respect to that obtained at 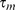 = 10 ms in case of C 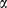-C 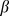, C 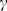-CO, C 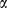-C 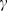, C 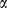-CO, and C 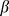-CO cross-peaks, and at 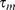 = 20 ms for C 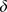-C 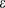 and C 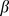-C 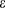 cross-peaks. All measurements were made at 30 kHz of MAS using PARIS-xy (m = 1)(N = 1/2) recoupling scheme and an RF amplitude of 30 kHz.

In Figure 10 magnetisation transfer improves by 7.3, 6.7, 16.4, and 16.0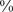 in case of C 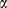-C 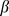, C 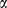-CO, C 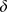-C 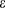, and C 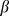-CO respectively upon changing 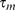 from 10 to 160 ms. The fact that both C 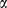-C 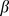 and C 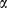-CO cross-peaks show nearly similar improvement tells us that 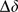 between the two carbons is not the main driving factor in this case. It seems that spatial distance between the two carbons is acting as a controlling factor here. This can be verified by comparing the build-up curves of a pair of chemically similar, but spatially dissimilar cross-peaks. C 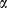-CO and C 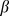-C 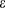 cross-peaks make one such pair. As expected, magnetisation improves by only 6.7 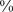 in case of C 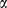-CO and by 23.5 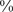 in case of C 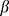-C 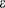 cross-peak. But if that is the case, C 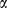-C 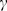 cross-peak should also follow a trend similar to C 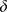-C 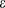 and C 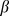-CO, which is not the case here. It shows a huge improvement of 65.5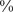. Similarly, C 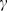-CO cross-peak also shows less improvement (41.9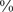) than C 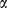-C 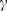 though the carbon nuclei involved are spatially more apart in the former. Hence, some other factors also seem to be contributing here apart from spatial distance. It is known that the efficiency of second-order recoupling schemes is strongly dependent on the strength of heteronuclear (^13^C- ^1^H) and homonuclear (^1^H- ^1^H) couplings, and thus has a complicated dependence on the nature of the system one is working with [7], [27]. Therefore, it is difficult to explain all the trends on the basis of only two factors, spatial distance and chemical-shift difference. But one can still say that spatial distance is one of the key factors in this case and longer mixing times are essential for observing long-range contacts.

## Materials and Methods

All solid-state NMR measurements were performed at fields of 11.74 and 16.43 T (corresponding to 125.76 and 176.07 MHz of ^13^C Larmor frequency, respectively) using Bruker AV and AVIII NMR spectrometers, respectively. 2.5 mm double- (at 11.74 T) and triple- (at 16.43 T) resonance probes were used to conduct experiments at a MAS frequency of 30 kHz. Commercially purchased U-^13^C-,^15^N-L-histidine

H
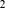
O and U-^13^C-,^15^N-fMLF were used for the experiments without any further purification. A sample of commercially available adamantane was used for the calibration of RF amplitude via nutation experiments.

Solid-phase peptide synthesis was employed to synthesise a 42 amino acid long Amyloid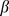 (A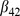) peptide according to 9-fluorenylmethoxycarbonyl (Fmoc) protocol. The peptide was isotopically labelled at specific positions using uniformly ^13^C- and ^15^N-labelled amino acids Gln15, Phe19, Ala30, Leu34, Val36, and Gly38 and uniformly ^15^N labeled His13. Synthesis products were purified to 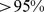 by HPLC as confirmed by mass spectrometry. To obtain amyloid fibrils of A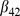, a 400 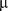
M solution of purified peptide in HEPES (4-(2-hydroxyethyl)-1-piperazineethanesulfonic acid) buffer (pH = 7.4) was kept at room temperature with mild rotation (10 rpm) for 4 days. Fibril formation was confirmed by electron microscopy.

Cross polarisation to ^13^C from ^1^H was implemented using a linearly ramped RF amplitude [37] centered around 72 kHz on the ^1^H channel with simultaneous application of RF field with an amplitude of 47 kHz on the ^13^C channel for 2.5 ms. ^1^H dipolar decoupling was applied in both dimensions using SW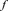-TPPM decoupling scheme [38] with an RF amplitude of 115 kHz on the ^1^H channel. A 2 s recycle delay was used in between scans everywhere. 2D ^13^C-^13^C spectra of histidine and N-fMLF were recorded by acquiring 128 points in the 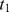 dimension with a dwell time of 11.4 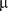
s. A total of 16 scans per free induction decay (FID) was recorded. 2D ^13^C-^13^C spectra of A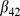 were recorded by acquiring 256 points in the 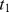 dimension with a dwell time of 11.4 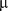
s and 256 scans per FID were recorded.

All data were processed and analysed using TOPSPIN 1.3. 2D data of histidine, N-fMLF, and A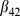 were zero filled in the 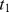 and 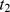 dimensions to 512 and 4096 points respectively. A mixed sine/cosine squared (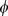 = 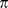/3 at 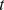 = 0) apodisation function was used in the 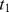 dimension for histidine and N-fMLF, and in both dimensions in case of A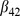.

## Conclusions

The detailed observations listed here may be summarised as follows with regard to two distinct magnetic fields.

Recoupling schemes of choice at 11.74 T for efficient magnetisation transfer among:

- Chemically close ^13^C nuclei: Recoupling schemes with half-rotor-synchronised pulses i.e. PARIS (N = 1/2), PARIS-xy (m = 1)(N = 1/2) and PARIS-xy (m = 2)(N = 1/2) should be used. Magnetisation transfer efficiency of these schemes drops as the RF amplitude is increased. Low RF amplitudes (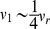) lead to much better transfer, and hence, should be applied.
- Chemically apart and far-apart ^13^C nuclei: Either schemes containing two-rotor-synchronised pulses (i.e. PARIS (N = 2) and PARIS-xy (m = 1)(N = 2) or PARIS-xy (m = 1)(N = 1/2) should be used. RF amplitude as low as 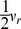 is sufficient for efficient magnetisation transfer, and hence, should be employed.

PARIS-xy (m = 1)(N = 1/2) scheme when employed using an RF amplitude of nearly half the MAS frequency is mostly efficient in transferring magnetisation among all kind of ^13^C nuclei. Hence, this should be the sequence of choice for performing 2D ^13^C-^13^C correlation experiments at moderately high magnetic fields and MAS frequencies.

Recoupling schemes of choice at 16.43 T for efficient magnetisation transfer among:

- Chemically close ^13^C nuclei: Recoupling schemes containing half-rotor-synchronised pulses i.e. PARIS (N = 1/2), PARIS-xy (m = 1)(N = 1/2) and PARIS-xy (m = 2)(N = 1/2) should be used. Low RF amplitudes (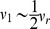) are preferable and lead to much better magnetisation transfer.
- Chemically apart and far-apart ^13^C nuclei: Recoupling schemes containing two-rotor-synchronised pulses i.e. PARIS (N = 2) and PARIS-xy (m = 1)(N = 2) should be used. High RF amplitudes (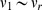) are required for efficient magnetisation transfer though.

None of the schemes studied in this work is capable of efficient magnetisation transfer among all kinds of ^13^C nuclei using a unique RF amplitude at this high magnetic field. A way out of this situation is recording a set of two complementary spectra using (1) PARIS-xy (m = 2)(N = 1/2) scheme and low RF amplitude (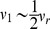) for obtaining intense cross-peaks belonging to class A and (2) PARIS-xy (m = 1)(N = 2) scheme and a slightly higher RF amplitude for obtaining intense cross-peaks belonging to class B and C. It is also advised to record a third spectrum using PARIS-xy (m = 2)(N = 1/2) scheme with 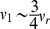. This will yield relatively weak cross-peaks but among all kind of ^13^C nuclei, and hence, can be quite helpful whilst assigning chemical shifts.
